# Identification of a Five-Gene Panel to Assess Prognosis for Gastric Cancer

**DOI:** 10.1155/2022/5593619

**Published:** 2022-02-09

**Authors:** Shuxin Li, Qianqian Mao, Zixuan Zhang, Yuqi Wang, Duoxuan Chen, Zhenwen Chen, Jianyi Lu

**Affiliations:** School of Basic Medical Sciences, Capital Medical University, Beijing Key Laboratory of Cancer Invasion & Metastasis Research, Beijing, China

## Abstract

**Methods:**

Two datasets were used as training and validation cohorts to establish the predictive model. We used three types of screening criteria: background analysis, pathway analysis, and functional analysis provided by the cBioportal website. Fisher's exact test and multivariable logistic regression were performed to screen out related genes. Furthermore, we performed receiver operating characteristic (ROC) and Kaplan–Meier curve analyses to evaluate the correlation between the selected genes and overall survival.

**Result:**

We screened five genes (KNL1, NRXN1, C6, CCDC169-SOHLH2, and TTN) that were highly related to recurrence of GC. The area under the receiver operating characteristic (ROC) curve was 0.813, which was much higher than that of the baseline model (AUC = 0.699). This result suggested that the mutation of five selected genes had a significant effect on the prediction of recurrence compared with other factors (age, stages, history, etc.). Furthermore, the Kaplan-Meier estimator also revealed that the mutation of five genes positively correlated with patient survival.

**Conclusions:**

The patients who have mutations in these five genes may experience longer survival than those who do not have mutations. This five-gene panel will likely be a practical tool for prognostic evaluation and will provide another possible way for clinicians to determine therapy.

## 1. Introduction

Gastric cancer, also known as stomach cancer, is one of the most malignant tumors worldwide and is still a major health threat in Asia-Pacific regions [1]. Evidence has shown that approximately 10% of stomach cancers have familial clustering. Genome-wide association studies have implicated the prostate stem cell antigen (PSCA) gene and the mucin1 (*MUC1*) gene as influencing susceptibility [2]. With high-resolution SNP arrays, researchers identified 22 recurrent genomic alterations, such as *FGFR2*, *ERBB2*, *KLF5*, and *GATA6* [3]. These results suggest that some key genes are involved in pathological progression. Up to 50% of advanced stage GC patients have peritoneal metastasis, which is also a sign of recurrence [4]. The recurrence rate of GC is approximately 42%, and the median survival time is 11-12 months [5]. Early detection of recurrence will significantly improve the prognosis of GC. Although harboring high precision, there is also a lag effect [6]. Moreover, overestimation of recurrence will unnecessarily increase the medical cost. Considering these factors, it is necessary to explore a plausible and practical way to assess the possibility of gastric cancer recurrence.

Tumorigenesis is a multistep process in which many somatic mutations are involved. Most mutations are random and probably occur as the cancer develops [7]. However, a subset of a few hundred genes is presumed to be involved in neoplasia progression and has been mutated at high frequency. These genes are referred to as driver genes, whose mutations tune gene expression towards specific tumor evolution [8]. Deep mining from tumor genomic profiling and searching for driver genes are helpful to understand the molecular mechanism of tumorigenesis and provide guidance for the prevention, treatment, and prognosis of patients.

Recently, due to the prevalence of next-generation sequencing technology, many research groups have performed tumor-related sequencing analysis [9, 10]. For resource integration and efficient utilization, The Cancer Genome Atlas (TCGA) and the International Cancer Genome Consortium (ICGC) datasets are established and provide researchers with a convenient way to obtain the entire sequence signature of cancer cases [11, 12]. To detect candidate tumor driver genes, many algorithms have been developed according to different principles. The main algorithm principles for driver gene identification are grouped into five categories: a single gene mutation frequency with the entire genome background mutation rate [13, 14], the effect of the mutant gene on biological function [15, 16], biological network or pathway analysis [17, 18], and data integration-based analysis [19, 20]. However, each algorithm has limitations or biases. For instance, classical mutation frequency-based approaches often have false-positive discoveries owing to tumor heterogeneity and other factors [21]. The network is often error prone because it is based on large-scale experimental data or computational prediction data [22]. It is plausible to combine various approaches to screen out driver genes and improve accuracy.

In the present study, we selected nine different algorithms based on the above principles to identify potential driver genes of gastric cancer based on DNA sequencing data from the TCGA-STAD project [23–25]. Then, we analyzed the correlation between the selected genes and the recurrence of patients. Five mutated genes, *KNL1*, *NRXN1*, *C6*, *CCDC169-SOHLH2*, and *TTN*, showed a significant negative correlation with the recurrence of gastric cancer through multivariable logistic regression analysis.

In summary, our study constructed a five-gene panel to predict the prognosis of gastric cancer. This study can provide new insights into the molecular mechanism of gastric cancer and a theoretical basis for precision medicine.

## 2. Materials and Methods

### 2.1. Cancer Sequencing Data

We used all DNA sequencing data and clinicopathological information from the TCGA Data Portal (https://portal.gdc.cancer.gov). We used data from 229 patients enrolled in the TCGA-STAD project as training cohort and 440 samples in the TCGA-PanCancer Atlas and 22 samples from a manuscript by Wang et al. [26] as validation cohort.

### 2.2. Workflow

**Figure 1:**
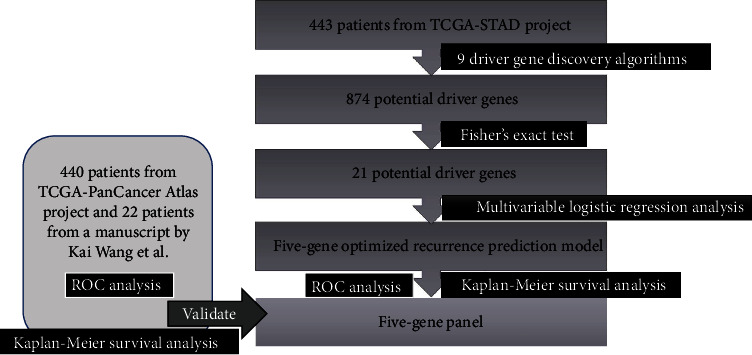
Workflow of our analysis. Flow chart showed the workflow of our present analysis.

The basic workflow for data analyses was described in a previous study and is listed in Figure 1 [27]. First, we downloaded the genomic DNA sequencing data of 229 patients with gastric cancer from the TCGA-STAD project. Second, potential cancer driver genes were identified from these data using nine driver gene discovery algorithms. We found 875 potential driver genes in total. Then, we made a Venn diagram to identify 159 genes that overlapped with each other as potential driver genes. Next, we used Fisher's exact test to detect the association of potential driver genes with the recurrence of gastric cancer. We found 21 potential driver gene (*KRAS*, *TSPOAP1*, *C6*, *CCDC169-SOHLH2*, *DNAH9*, *MAP7D1*, *NCKAP5*, *NRXN1*, *PREX2*, *SMG1*, *TNKS1BP1*, *TTN*, *ABCB4*, *ALK*, *ATXN1*, *ASTN2*, *C2ORF16*, *CARD6*, *KNL1*, *CENPF*, *CLCNKA*) in this step. The statistically significant genes were then subjected to multivariable logistic regression analysis to construct a recurrence prediction model. We obtained five genes in this step, which were the final genes we identified in the five-gene panel. Receiver operating characteristic (ROC) analysis and Kaplan-Meier survival analysis were used to verify the reliability of the five-gene panel in predicting recurrence.

### 2.3. Identification of Gastric Cancer Driver Genes

**Figure 2:**
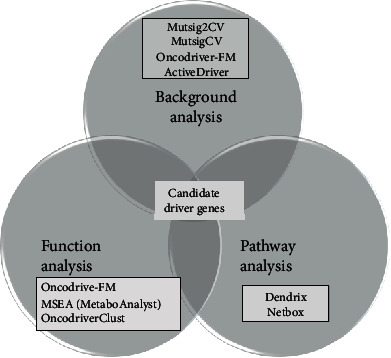
The Venn diagram of selecting driver genes. The Venn diagram shows the process we used to screen out the driver genes through three types of analysis tools.

The DNA sequencing data of the patients enrolled in the TCGA-STAD project were used to identify potential driver genes using nine algorithms based on three theories, including mutation frequency differences or background differences, functional impacts, and pathway or network enrichment. We first used the Musig2CV, OncodriverFM, and ActiveDriver algorithms [27], which are based on the mutation frequency of an individual gene compared with the background mutation rate. Then, we used structural genomic-based algorithms that identified driver genes with the characteristics of mutual exclusivity and incorporated copy number variation (CNV) data for driver gene identification, including Dendrix, MSEA, OncodriveCLUST, and pathway analysis algorithms, including Dendrix and Netbox. The detailed criteria of each method used to identify driver genes are listed in Table 1 [27]. Then, to improve the accuracy of the results, we used a Venn diagram to select the potential driver genes detected in at least three algorithms described as Figure 2.

### 2.4. Developing the Recurrence Prediction Model

To illustrate the mutational landscape between the recurrence and the growth of new tumor vs. the recurrence-free group, we carried out Fisher's exact test. To develop an optimized recurrence prediction model, the recurrence-associated genes identified above and the patients' clinicopathological information were subjected to multivariable logistic regression analysis. The model was evaluated using ROC analysis [28]. Additionally, we performed Kaplan-Meier survival analysis to evaluate clinical significance [29].

### 2.5. Statistical Analysis

To detect the association of potential driver genes with the recurrence of gastric cancer, we used Fisher's exact test. To construct a recurrence prediction model, we performed a multivariable logistic regression analysis. To assess the sensitivity and specificity of the recurrence models, we conducted an ROC analysis and calculated the AUC. To estimate the prognosis, we performed Kaplan-Meier survival analysis. A *p* value of less than 0.05 was considered statistically significant, and IBM SPSS Statistics 22 Software was used for all the statistical analyses.

## 3. Results

### 3.1. Clinical Characteristics of Patients with Gastric Cancer

To identify the driver genes of gastric cancer, we searched for the genome sequencing data of 443 patients with gastric cancer obtained from the TCGA-STAD Data Portal (stomach adenocarcinoma, TCGA, provisional). After removing the data for which new tumor events were not available, we had 229 patients in total.  Table 2 shows the pathological and clinical characteristics of the patients. High-grade tumors comprised 57.7% of the analysis cohort, whereas low-grade tumors comprised 42.3%. Among 57.7% patients with the available recurrence records, 48 patients (21.0%) relapsed with new tumor events, while 181 patients (79.0%) had recurrence-free tumors.

### 3.2. Differential Mutational Landscape in the Gastric Cancer Recurrence Cohort vs. the Recurrence-Free Cohort

We performed nine algorithms based on three theories, including mutation frequency differences or background differences, functional impacts, and pathway or network enrichment, and selected 159 potential driver genes screened out by at least three different algorithms. The 159 genes that we selected were considered to be potential driver mutations in gastric cancer. Next, we divided 229 patients with known recurrence records into two cohorts based on the presence (*n* = 181) vs. the absence of disease recurrence (*n* = 48) to obtain the general recurrence rate. The recurrence of patients harboring mutations in each potential driver gene was also calculated. We found 21 potential driver genes (*KRAS*, *TSPOAP1*, *C6*, *CCDC169-SOHLH2*, *DNAH9*, *MAP7D1*, *NCKAP5*, *NRXN1*, *PREX2*, *SMG1*, *TNKS1BP1*, *TTN*, *ABCB4*, *ALK*, *ATXN1*, *ASTN2*, *C2ORF16*, *CARD6*, *KNL1*, *CENPF*, *CLCNKA*) using Fisher's exact test, which were significantly enriched in the recurrence-free group and were negatively associated with gastric cancer recurrence (Table 3).

### 3.3. Development of the Five-Gene Diagnostic Panel for Gastric Cancer

According to the principle of Fisher's exact test, we sorted the 21 genes by *p* value from minimum to maximum and selected ten genes that had the lowest *p* values. Next, multivariable logistic regression analysis was performed to construct a diagnostic model based on pathological and clinical information of the patients (*n* = 229). Five genes were significantly associated with gastric cancer recurrence or new tumor events by multivariable logistic regression. Additionally, the patient's age, gender, race, tumor stage, tumor grade, and family history of cancer were regarded as the independent variables, and the state of recurrence was the dependent variable. Finally, an optimized recurrence prediction model was constructed using the logistic regression equation:

(1) $$\begin{array}{c} \text{logit}\left(\text{P}\right)=31.641-16.884\times \text{age}\left(30-50\right)-16.057\times \text{age}\left(50-70\right)–15.828\times \text{age}\left(70-90\right)–12.896\times \text{grade}1-13.936\times \text{grade}2–13.767\times \text{grade}3+0.471\times \text{stageI}+0.884\times \text{stageII}+0.294\times \text{stageIII}-1.284\times \text{no family history of stomach cancer}-0.659\times \text{White}+0.821\times \text{Asian}-19.562\times \text{Black}-0.961\times \text{male}–2.014\times \text{KNL}1–1.143\times \text{TTN}–1.260\times \text{NRXN}1-0.517\times \text{CCDC}169-\text{SOHLH}2–2.178\times \text{C}6 \end{array}$$

The Exp (*B*) values for the four genes (KNL1, NRXN1, C6, TTN) were all less than 0.5, indicating that these genes might significantly decrease the probability of recurrence. The *B* value of CCDC169-SOHLH2 is -0.517, and its Exp (*B*) value is 0.596, indicating that the mutation of CCDC169-SOHLH2 might decrease the recurrence of GC, though this is less obvious than the other genes. However, we chose to use it. It was reported that KNL1 was upregulated in GC tissues and contributed to the proliferation of cancer cells [30]. The Exp (*B*) of NRXN1 is 0.284, which was also reported to be closely associated with gastric cancer [31]. Supported by the research data, we obtained a 5-gene (*KNL1*, *NRXN1*, *C6*, *CCDC169-SOHLH2*, *TTN*) prognostic panel for further evaluation.  Table 4 shows the multivariable logistic regression analysis of variables for establishing the recurrence prediction model.

### 3.4. Prognostic Value of the Five-Gene Recurrent Prediction Model

**Figure 3:**
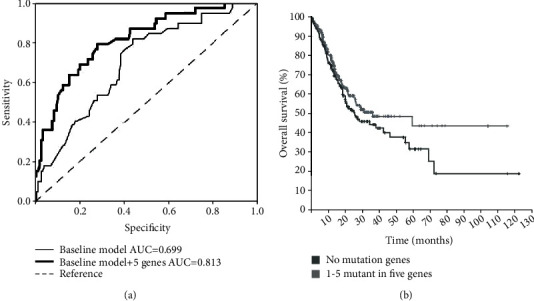
Recurrence prediction with ROC analysis and Kaplan-Meier survival analysis with the five-gene panel in the TCGA-STAD project. (a) Recurrence prediction with ROC (receiver operating characteristic) analysis in the TCGA-STAD project. The baseline model was established according to the patient's age, tumor stage, grade, gender, and race, and ROC curve was generated accordingly. The AUC (area under curve) was 0.699. After adding the five-gene prognostic panel to the baseline model, the AUC was 0.813, and the *p* value was 0.007. (b) Kaplan-Meier survival analysis with the five-gene prognostic panel in the TCGA-STAD project. Survival of patients with at least one mutation in the five genes (no less than one gene) showed significant longer survival time than those without mutations in these five genes (no mutant genes), *p* value =0.198.

Diagnostic tests are often evaluated by some parameters, such as sensitivity and specificity. Such evaluation is an essential step towards developing a test with desirable levels of sensitivity and specificity. The area under the ROC curve (AUC) is a global measure of a test to discriminate whether a specific condition is present [32]. Here, we performed ROC analysis to assess our recurrence prediction model. ROC curves were established on the baseline model according to the patients' age at initial diagnosis, gender, tumor stage, tumor grade, and race. The AUC of the baseline model was 0.699 (Figure 3(a)). Since all the patients had at least one mutation of the five genes, we added the five genes to the baseline model and found that the AUC rose to 0.813 as expected (*p* < 0.01). This result suggested that the five-gene panel greatly improved the credibility of the prediction model.

### 3.5. Survival Analysis of the Five-Gene Panel in Gastric Cancer Cohorts

Furthermore, we performed Kaplan-Meier survival analysis to evaluate the effects of mutations in five genes on the prognosis of GC patients. As shown in Figure 3(b), the overall survival time of the patients with mutations in either of these five genes was significantly longer than that without any mutations in these five correlated genes. This result indicated that mutations of these genes were significantly related to better prognosis.

### 3.6. Validation of the Prognostic Panel in two databases

**Figure 4:**
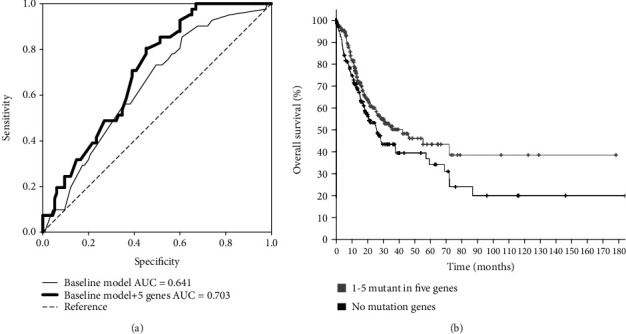
The verified ROC curve and Kaplan-Meier survival analysis. (a) Recurrence prediction with ROC (receiver operating characteristic) analysis in the validation cohort. The baseline model was established according to the patient's age, tumor stage, grade, gender, and race. The AUC (area under curve) was 0.641. After adding the five-gene prognostic panel to the baseline model, the AUC was 0.703, and the *p* value was less than 0.05. (b) Kaplan-Meier survival analysis with the five-gene prognostic panel in the validation cohort. Survival of patients with at least one mutation in the five genes showed significant longer survival time than those without mutations in these five genes (no mutant genes), *p* < 0.05.

To investigate the applicability of the five-gene panel in predicting the recurrence of GC, we combined another two data sets: TCGA gastric cancer cohort, which consists of 440 mutation data and clinical data collected in TCGA PanCancer Atlas and 22 exome sequencing data from GC patients [26]. According to the method we mentioned above, a baseline model was also constructed with the patient's age, tumor stage, gender, and race. The ROC curve is shown in Figure 4(a), and the AUC of baseline is 0.641. Then, we added all five genes to the baseline model, and the AUC was 0.703 (*p* < 0.05). This verified the five-gene panel reliability. We also carried out Kaplan-Meier survival analysis. As shown in Figure 4(b), patients with mutations in any of the five genes survived significantly better than those who did not (*p* < 0.05).

## 4. Discussion

Gastric cancer currently ranks as the fifth most diagnosed cancer and the third leading cause of cancer death [33]. Because of its insidious onset, it is very often diagnosed at an advanced stage, and prognoses are still unsatisfactory due to the high incidence of recurrence [34]. At present, GC markers have been used for diagnosis, determination of clinical stage, and evaluation of treatment responses. CEA and CA199 are routinely recommended in clinical practice. However, serum tumor biomarkers have limitations due to insufficient specificity and sensitivity. In recent years, next-generation sequencing (NGS) technology has been widely used to screen out tumor biomarkers, which contribute to the dynamic observation of tumorigenesis and development, clinical efficacy, and prognosis evaluation. The molecular features of gastric cancer are multifaceted and heterogeneous, such as chromosomal instability, microsatellite instability, microRNA deregulation, somatic gene mutations, or functional single nucleotide polymorphisms [23]. Wang et al. performed whole-genome sequencing in 100 tumor-normal pairs for integrative genomic analysis and identified previously known (TP53, ARID1A, and CDH1) and new (MUC6, CTNNA2, GLI3, RNF43, and others) significantly mutated driver genes [24]. Although some studies reported that FGFR2 was overexpressed in 31.1% of GC patients and might be associated with vascular invasion, FGFR2 amplification enhanced the sensitivity of regorafenib in gastric cancer and colorectal cancer [35, 36]. Patients with somatic CDH1 epigenetic and structural alterations have worse overall survival than those without alterations [37]. Although the frequency of mutated genes is relatively low, they have a great impact on patients when considered together. It is clear that the gene mutation signature improves the diagnostic accuracy, therapeutic strategy, and prognostic judgment.

GC has a relatively high relapse rate. A retrospective study showed that recurrence occurred in 20.5% of patients [38]. The development of a precise evaluation of recurrence risk is important to reduce overtreatment and achieve satisfactory outcomes. Genome-wide analysis has allowed characterization on a genomics basis and found many potential driver genes in GC [39–41]. In the present study, we analyzed the DNA sequencing data of 229 patients from the TCGA-STAD project and identified five potential driver genes (*CCDC169-SOHLH2*, *TTN*, *KNL1*, *C6*, *NRXN1*) whose mutations were negatively associated with gastric cancer recurrence (*p* < 0.01). These five genes are all related to cancer pathological processes according to previous reports. Among them, Sohlh2 was demonstrated to be an important inhibitor of ovarian cancer cell proliferation and metastasis by repressing the MMP9 expression [42]. Sohlh2 also suppressed breast cancer cell proliferation through Wnt signaling [43]. TTN is one of the most frequently mutated genes in GC [44]. Nonsynonymous mutations in TTN were found in its coding regions in different cancer types, half of which might be considered driver mutations [45]. According to a correlation analysis of lung cancer, missense mutation of TTN may indicate good prognosis [46]. Evidence has shown that KNL1 plays an effective role in decreasing apoptosis and promoting the proliferation of colorectal cancer cells, and downregulation of KNL1 by miR-193b-3p significantly induces cell differentiation [47]. A recent report developed a novel pathway and reach (PAR) method and identified 50 candidate driver genes, among which C6 ranked in the top five [48]. A comprehensive survey of genomic alterations in GC revealed that C6 was a recurrent neoantigen [49]. These findings confirm our findings. In GC, NRXN1 is one of the altered genes significantly related to mutated TP53, and NRXN1 mutation is significantly associated with different drug responses [31].

In the present study, we constructed a recurrence prediction model with five recurrence-associated genes through multivariable logistic regression analysis. This allowed us to determine the effect of each factor. The data showed that any mutation in the five genes is negatively related to recurrence. The AUC was 0.699 in a baseline model based on age, gender, tumor stage, tumor grade, family history of cancer, and race as independent variables. The five-gene prognostic panel increased the AUC to 0.813 (*p* < 0.01). Moreover, the Kaplan-Meier survival analysis curve also revealed that patients with any mutation of these five genes in this panel had better survival time. Furthermore, we verified the panel on a TCGA-PanCancer Atlas Project dataset and research performed by Wang et al. [26] and obtained a consistent conclusion. This indicates that this five-gene panel may have potential application value.

Although performed on two cohorts, there are several limitations of our analysis. Because of the lack of recurrence information for some patients, it is difficult to validate this five-gene panel in larger datasets. Additionally, the gene panel generated from our analysis may vary considerably among individual studies. Therefore, it is essential to detect its accuracy before its development as a biomarker for GC recurrence.

In conclusion, we constructed a five-gene panel as a prognostic factor to predict the recurrence of patients with gastric cancer based on data from TCGA. Further studies are needed to evaluate the availability of the gene panel. This panel is helpful for reducing treatment cost and facilitating better cancer management.

## Figures and Tables

**Table 1:** Driver gene discovery algorithms.

Algorithms	Criteria
Active driver	FDR < 0.05
MutSigCV	FDR < 0.05
MutSig2CV	FDR < 0.1
Oncodrive-FM	FDR < 0.05
MSEA	*p* value <0.05
OncodriveClust	FDR < 0.05
Dendrix	Genes in at least 10% of modules in any *K*
Netbox	Genes included in all the modules
DriverNet	*p* value <0.01

**Table 2:** Demographics and tumor characteristics of patients with gastric cancer in TCGA-STAD project.

Characteristics	Number of patients	Percent (%)
Gender		
Male	175	67.2
Female	54	32.8
Race		
Asian	69	30.1
White	135	58.9
Black or African American	10	4.4
Unknown	15	6.6
Family cancer history		
Yes	14	7.0
No	185	93.0
New tumor event		
Yes	48	21.0
No	181	79.0
Tumor grade		
1	7	3.1
2	90	39.3
3	127	55.4
4	5	2.2
Pathological tumor stage		
Stage I	30	13.5
Stage II	65	29.3
Stage III	107	48.2
Stage IV	20	9.0
Age at initial pathologic diagnosis		
30-50 (including 30)	16	8.8
50-70 (including 50)	94	51.6
70-90 (including70)	71	39.0
90+ (including 90)	1	0.6

**Table 3:** Potential driver genes significantly associated with gastric cancer recurrence and verified by Fisher's exact test.

Gene name	Cytogenetic band	Description	Ensembl ID	*p* values
TTN	2q31.2	Titin	ENSG00000155657	0.0001441
KNL1	15q15.1	Kinetochore scaffold 1	ENSG00000137812	0.005038
C6	5p13.1	Complement C6	ENSG00000039537	0.01131
NRXN1	2p16.3	Neurexin 1	ENSG00000179915	0.01192
CCDC169-SOHLH2	13q13.3	Spermatogenesis and oogenesis specific basic helix-loop-helix 2 coiled-coil domain containing 169	ENSG00000250709	0.01255
KRAS	12p12.1	KRAS protooncogene, GTPase	ENSG00000133703	0.02746
TSPOAP1	17q22	TSPO-associated protein 1	ENSG00000005379	0.03608
DNAH9	17p12	Dynein axonemal heavy chain 9	ENSG00000007174	0.03468
MAP7D1	1p34.3	MAP7 domain containing 1	ENSG00000116871	0.03282
NCKAP5	2q21.2	NCK-associated protein 5	ENSG00000176771	0.02106
PREX2	8q13.2	Phosphatidylinositol-3,4,5-trisphosphate-dependent Rac exchange factor 2	ENSG00000046889	0.01929
SMG1	16p12.3	SMG1 nonsense mediated mRNA decay associated PI3K-related kinase	ENSG00000157106	0.03282
TNKS1BP1	11q12.1	Tankyrase 1 binding protein 1	ENSG00000149115	0.01931
ABCB4	7q21.12	ATP binding cassette subfamily B member 4	ENSG00000005471	0.02506
ALK	2p23.2-p23.1	ALK receptor tyrosine kinase	ENSG00000171094	0.02106
ATXN1	6p22.3	Ataxin 1	ENSG00000124788	0.0488
ASTN2	9q33.1	Astrotactin 2	ENSG00000148219	0.01718
C2ORF16	2p23.3	Chromosome 2 open reading frame 16	ENSG00000221843	0.02144
CARD6	5p13.1	Caspase recruitment domain family member 6	ENSG00000132357	0.0488
CLCNKA	1p36.13	Chloride voltage-gated channel Ka	ENSG00000186510	0.03672
CENPF	1q41	Centromere protein F	ENSG00000117724	0.01131

**Table 4:** Multivariable logistic regression analysis of variables for establishing the recurrence prediction model.

	*B*	SE	Wald	Df	Sig	Exp (*B*)	95% CI for Exp (*B*)	
							Lower	Upper
Genes								
TTN	-1.143	.484	5.579	1	.018	.319	.123	.823
KNL1	-2.014	.920	4.788	1	.029	.133	.022	.811
NRXN1	-1.260	.698	3.253	1	.071	.284	.072	1.115
CCDC169-SOHLH2	-.517	.913	.321	1	.571	.596	.100	3.569
C6	-2.178	.767	8.059	1	.005	.113	.025	.510
Clinical characteristics								
Grade 1	-12.896	2383.604	.000	1	.996	2.508*E*-6	.000	.c
Grade 2	-13.936	2383.604	.000	1	.995	8.861*E*-7	.000	.c
Grade 3	-13.767	2383.604	.000	1	.995	1.050*E*-6	.000	.c
Stage I	.471	1.088	.187	1	.665	1.601	.190	13.511
Stage II	.884	1.003	.776	1	.378	2.420	.339	17.294
Stage III	.294	.921	.102	1	.750	1.342	.221	8.160
Age 30-50	-16.884	.778	470.483	1	.000	4.647*E*-8	1.011*E*-8	2.137*E*-7
50-70	-16.057	.500	1029.232	1	.000	1.064*E*-7	3.988*E*-8	2.836*E*-7
70-90	-15.828	.000	.	1	.	1.336*E*-7	1.336*E*-7	1.336*E*-7
White	-.659	1.063	.384	1	.535	.518	.064	4.154
ASINA	.821	1.195	.472	1	.492	2.273	.218	23.646
Black or African American	-19.562	6066.729	.000	1	.997	3.195*E*-9	.000	.c
Male	-.961	.551	3.046	1	.081	.383	.130	1.125
History	1.284	.759	2.858	1	.091	3.610	.815	15.997

## Data Availability

Previously reported DNA sequencing data were used to support this study and are available at TCGA Data Portal (https://portal.gdc.cancer.gov) and cbioportal (https://www.cbioportal.org/).

## References

[1] Xiao S., Zhou L. (2017). Gastric cancer: metabolic and metabolomics perspectives (review). *International Journal of Oncology*.

[2] McGuire S. (2016). World Cancer Report 2014. Geneva, Switzerland: World Health Organization, International Agency for Research on Cancer, WHO Press, 2015. *Advances in Nutrition*.

[3] Deng N., Goh L. K., Wang H. (2012). A comprehensive survey of genomic alterations in gastric cancer reveals systematic patterns of molecular exclusivity and co-occurrence among distinct therapeutic targets. *Gut*.

[4] Mikula-Pietrasik J., Uruski P., Tykarski A. (2018). The peritoneal "soil" for a cancerous "seed": a comprehensive review of the pathogenesis of intraperitoneal cancer metastases. *Cellular and Molecular Life Sciences*.

[5] Bang Y. J., Van Cutsem E., Feyereislova A. (2010). Trastuzumab in combination with chemotherapy versus chemotherapy alone for treatment of HER2-positive advanced gastric or gastro-oesophageal junction cancer (ToGA): a phase 3, open-label, randomised controlled trial. *Lancet*.

[6] Virmani V., Khandelwal A., Sethi V. (2012). Neoplastic stomach lesions and their mimickers: spectrum of imaging manifestations. *Cancer Imaging*.

[7] Waks Z., Weissbrod O., Carmeli B., Norel R., Utro F., Goldschmidt Y. (2016). Driver gene classification reveals a substantial overrepresentation of tumor suppressors among very large chromatin-regulating proteins. *Scientific Reports*.

[8] Hausser J., Alon U. (2020). Tumour heterogeneity and the evolutionary trade-offs of cancer. *Nature Reviews. Cancer*.

[9] Wang Z., Jensen M. A., Zenklusen J. C. (2016). A practical guide to the cancer genome atlas (TCGA). *Methods in Molecular Biology*.

[10] Zhang J., Bajari R., Andric D. (2019). The international cancer genome consortium data portal. *Nature Biotechnology*.

[11] Kunimasa K., Hirotsu Y., Amemiya K. (2020). Genome analysis of peeling archival cytology samples detects driver mutations in lung cancer. *Cancer Medicine*.

[12] Slavin T., Neuhausen S. L., Rybak C. (2017). Genetic gastric cancer susceptibility in the international clinical cancer genomics community research network. *Cancer Genetics*.

[13] Dees N. D., Zhang Q., Kandoth C. (2012). MuSiC: identifying mutational significance in cancer genomes. *Genome Research*.

[14] Tamborero D., Gonzalez-Perez A., Lopez-Bigas N. (2013). OncodriveCLUST: exploiting the positional clustering of somatic mutations to identify cancer genes. *Bioinformatics*.

[15] Sim N. L., Kumar P., Hu J., Henikoff S., Schneider G., Ng P. C. (2012). SIFT web server: predicting effects of amino acid substitutions on proteins. *Nucleic Acids Res*.

[16] Adzhubei I. A., Schmidt S., Peshkin L. (2010). A method and server for predicting damaging missense mutations. *Nature Methods*.

[17] Bashashati A., Haffari G., Ding J. (2012). DriverNet: uncovering the impact of somatic driver mutations on transcriptional networks in cancer. *Genome Biology*.

[18] Ng S., Collisson E. A., Sokolov A. (2012). PARADIGM-SHIFT predicts the function of mutations in multiple cancers using pathway impact analysis. *Bioinformatics*.

[19] Akavia U. D., Litvin O., Kim J. (2010). An integrated approach to uncover drivers of cancer. *Cell*.

[20] Bertrand D., Chng K. R., Sherbaf F. G. (2015). Patient-specific driver gene prediction and risk assessment through integrated network analysis of cancer omics profiles. *Nucleic Acids Research*.

[21] Lawrence M. S., Stojanov P., Polak P. (2013). Mutational heterogeneity in cancer and the search for new cancer-associated genes. *Nature*.

[22] Lee D., Gorkin D. U., Baker M. (2015). A method to predict the impact of regulatory variants from DNA sequence. *Nature Genetics*.

[23] Comprehensive molecular characterization of gastric adenocarcinoma (2014). Comprehensive molecular characterization of gastric adenocarcinoma. *Nature*.

[24] Wang K., Yuen S. T., Xu J. (2014). Whole-genome sequencing and comprehensive molecular profiling identify new driver mutations in gastric cancer. *Nature Genetics*.

[25] Kakiuchi M., Nishizawa T., Ueda H. (2014). Recurrent gain-of-function mutations of RHOA in diffuse-type gastric carcinoma. *Nature Genetics*.

[26] Wang K., Kan J., Yuen S. T. (2011). Exome sequencing identifies frequent mutation of ARID1A in molecular subtypes of gastric cancer. *Nature Genetics*.

[27] Han Y., Zheng Q., Tian Y. (2019). Identification of a nine-gene panel as a prognostic indicator for recurrence with muscle-invasive bladder cancer. *Journal of Surgical Oncology*.

[28] Linden A. (2006). Measuring diagnostic and predictive accuracy in disease management: an introduction to receiver operating characteristic (ROC) analysis. *Journal of Evaluation in Clinical Practice*.

[29] Goel M. K., Khanna P., Kishore J. (2010). Understanding survival analysis: Kaplan-Meier estimate. *Int J Ayurveda Res*.

[30] Song B., Du J., Song D. F. (2018). Dysregulation of NCAPG, KNL1, miR-148a-3p, miR-193b-3p, and miR-1179 may contribute to the progression of gastric cancer. *Biological Research*.

[31] Park S., Lee J., Kim Y. H., Park J., Shin J. W., Nam S. (2016). Clinical Relevance and Molecular Phenotypes in Gastric Cancer, of _TP53_ Mutations and Gene Expressions, in Combination With Other Gene Mutations. *Scientific Reports*.

[32] Hajian-Tilaki K. (2013). Receiver operating characteristic (ROC) curve analysis for medical diagnostic test evaluation. *Caspian Journal of Internal Medicine*.

[33] Bray F., Ferlay J., Soerjomataram I. (2018). Global cancer statistics 2018: GLOBOCAN estimates of incidence and mortality worldwide for 36 cancers in 185 countries. *CA: a Cancer Journal for Clinicians*.

[34] Wagner A. D., Syn N. L. X., Moehler M., Grothe W., Yong W. P., Tai B. C. (2010). Chemotherapy for advanced gastric cancer. *Cochrane Database of Systematic Reviews*.

[35] Nagatsuma A. K., Aizawa M., Kuwata T. (2015). Expression profiles of HER2, EGFR, MET and FGFR2 in a large cohort of patients with gastric adenocarcinoma. *Gastric Cancer*.

[36] Cha Y., Kim H. P., Lim Y., Han S. W., Song S. H., Kim T. Y. (2018). FGFR2 amplification is predictive of sensitivity to regorafenib in gastric and colorectal cancers in vitro. *Molecular Oncology*.

[37] Corso G., Carvalho J., Marrelli D. (2013). Somatic mutations and deletions of the E-cadherin gene predict poor survival of patients with gastric cancer. *Journal of Clinical Oncology*.

[38] Shin C. H., Lee W. Y., Hong S. W. (2016). Characteristics of gastric cancer recurrence five or more years after curative gastrectomy. *Chinese Journal of Cancer Research*.

[39] Xing R., Zhou Y., Yu J. (2019). Whole-genome sequencing reveals novel tandem-duplication hotspots and a prognostic mutational signature in gastric cancer. *Nature Communications*.

[40] Ong C. A., Shannon N. B., Ross-Innes C. S. (2014). Amplification of TRIM44: pairing a prognostic target with potential therapeutic strategy. *Journal of the National Cancer Institute*.

[41] Nagarajan N., Bertrand D., Hillmer A. M. (2012). Whole-genome reconstruction and mutational signatures in gastric cancer. *Genome Biology*.

[42] Dong M. M., Peng S. J., Yuan Y. N., Luo H. P. (2019). LncRNA TTN-AS1 contributes to gastric cancer progression by acting as a competing endogenous RNA of miR-376b-3p. *Neoplasma*.

[43] Zhang X., Liu R., Zhao N. (2019). Sohlh2 inhibits breast cancer cell proliferation by suppressing Wnt/*β*‐catenin signaling pathway. *Molecular Carcinogenesis*.

[44] Wang H., Shen L., Li Y. (2020). Integrated characterisation of cancer genes identifies key molecular biomarkers in stomach adenocarcinoma. *Journal of Clinical Pathology*.

[45] Greenman C., Stephens P., Smith R. (2007). Patterns of somatic mutation in human cancer genomes. *Nature*.

[46] Cheng X., Yin H., Fu J. (2019). Aggregate analysis based on TCGA: TTN missense mutation correlates with favorable prognosis in lung squamous cell carcinoma. *Journal of Cancer Research and Clinical Oncology*.

[47] Bai T., Zhao Y., Liu Y., Cai B., Dong N., Li B. (2019). Effect of KNL1 on the proliferation and apoptosis of colorectal cancer cells. *Technology in Cancer Research & Treatment*.

[48] Ramsahai E., Tripathi V., John M. (2019). Cancer driver genes: a guilty by resemblance doctrine. *PeerJ*.

[49] Chen C., Zhou Q., Wu R. (2019). A comprehensive survey of genomic alterations in gastric cancer reveals recurrent neoantigens as potential therapeutic targets. *BioMed Research International*.

